# Application of the Safe-by-Design Concept in Crop Breeding Innovation

**DOI:** 10.3390/ijerph17176420

**Published:** 2020-09-03

**Authors:** Jan Pieter van der Berg, Gijs A. Kleter, Evy Battaglia, Lianne M. S. Bouwman, Esther J. Kok

**Affiliations:** Wageningen Food Safety Research, Wageningen University and Research, Akkermaalsbos 2, P.O. Box 230, NL-6700 AE Wageningen, The Netherlands; gijs.kleter@wur.nl (G.A.K.); evy.battaglia@wur.nl (E.B.); lianne.bouwman@wur.nl (L.M.S.B.); leeuwe.kok@outlook.com (E.J.K.)

**Keywords:** crop breeding innovations, food, risk assessment, safe-by-design, synthetic biology

## Abstract

The present paper proposes the application of the safe-by-design concept to crop breeding innovation with the aim to accommodate safety considerations for new agricultural food and feed products. Safe-by-design can be implemented in all stages of the innovation cycle of agricultural products, from the early stages of research and development towards the post-market stage. Our proposed application of safe-by-design can be part of “responsible research and innovation” concepts, because they share features such as risk prevention strategies and a participatory approach. Early awareness of potential safety issues can guide the development of agricultural products towards safe options, both at the process and product level, and thus may help to reduce extensive pre-market assessment studies that might otherwise be needed further downstream for regulatory product approval. Here, it is discussed how the proposed safe-by-design approach can be introduced into the development of safe food crops using emerging technologies, such as gene editing and synthetic biology, and how this may help to safeguard the safety of our food and feed supply in the light of the ongoing global innovations in agricultural crop breeding.

## 1. Introduction

Safe-by-design, also known as “prevention through design” or “inherently safer design”, is a concept that aims to minimize the hazards of products, processes, and environments at an early phase of the design process. The initial focus of this concept entailed measures for the prevention of health risks, such as human accidents and illnesses, as well as environmental damage [1]. These preventive measures are applied at each stage of the design process, from the conceptualization to the finalization of the facility, process, or product. While known under different names, the idea of pro-active assessment and precautious prevention of risks has become commonplace and even enshrined in some countries’ legislation as a regulatory prerequisite in a wide range of sectors, such as construction, food and chemical manufacture, electric engineering, occupational health, and pharmaceuticals, amongst others (Table A1). For example, in 1969, the U.S. military adopted Military Standard #882 on “system safety”, describing an approach to eliminate hazards and reduce risks. Following a systematic approach, hazards are initially identified and the risks linked to these hazards are assessed, so that mitigation can be initiated to eliminate the hazard or otherwise reach the lowest risk level possible, according to a “hierarchy of controls” approach. In this approach, elimination or substitution of the (source of the) hazards is considered. If this is not possible, the options include the engineering of controls to remove the hazard from its source, administrative controls, warning systems, or protective measures to prevent exposure [2,3,4]. This approach has since become widely adopted throughout American industries [4].

Furthermore, in the pharmaceutical sector, “quality by design” of products has become enshrined in internationally harmonized guidelines. Under these guidelines, risk attributes of a product have to be identified at an early stage, and a risk management strategy has to be worked out, aiming to ensure product quality, from manufacture up until its “end of life”, as well as verification of the control strategy’s effectiveness [5]. Safety, in this context, is considered one of the quality aspects of a product.

The implementation of safe-by-design is particularly imperative in fields where governance may have difficulty in keeping pace with rapid technological innovations, as previously observed for nanomaterials [6]. We contend that a similar reasoning applies to crop breeding innovations based on emerging new breeding technologies (NBTs), primarily gene editing. These innovations have the potential to play a significant role in future agricultural genetically modified (GM) crops [7], but also raise new challenges due to possible changes in the physiology of edited plants that might pose potential hazards regarding food safety. In this respect, it is imperative to guarantee the safety of the resulting agricultural food and feed products, thus, maintaining the current high level of protection of human and animal health, as well as the environment. Potential socio-ethical implications, such as monopolisation, may also be associated with NBT innovations. Here, however, we will focus on potential food and feed safety issues.

For designing safe agricultural (GM) products using NBTs and their versatile applications, a novel approach is needed to effectively minimize hazards so as to ensure a safe end-product. Given that not all hazards associated with the use of these NBTs may be anticipated, certain unforeseen hazards, however remote, might pose risks that would, in the current system, only become apparent in the final stage of product development when the safety dossier is compiled. The prevention of hazards associated with a new product should therefore preferably be ensured through safety considerations at all stages of development, which is an essential feature of the safe-by-design concept.

The concept of safe-by-design has not yet been applied in agricultural biotechnology, but initial formats have been developed within Wageningen University and Research, which are currently being further explored in the Dutch research program on “Biotechnology and Safety” [8,9]. Recently, it was also proposed to introduce similar concepts into the fields of nanotechnology (i.e., NanoReg2; http://www.nanoreg2.eu) and synthetic biology [1]. These different fields have developed and adopted similar initial safe-by-design concepts that are based on the shared idea to design a product or process, which from the start has an intrinsically low risk potential, instead of applying protective measures to contain any potential risk.

The present paper discusses the proposed application of the safe-by-design principle, as an inherent part of a broader safety strategy, to crop breeding innovation, a field that is undergoing rapid transformation caused by the recent introduction of NBTs that make use of gene-editing techniques such as CRISPR Cas9. Our vision is that the application of the safe-by-design concept not only focuses on (bio)technological aspects of safe innovation, but also includes consultations with experts. We discuss how safe-by-design may also be part of a responsible research and innovation (RRI) strategy, which also focuses on ethical and societal aspects of research and development, and aims to align innovation with societal needs and expectations [10,11]. Furthermore, it will be discussed how the safe-by-design concept takes account of all potential safety aspects of new agricultural food and feed products, from the initial idea to the final product on the market, as well as the implementation of this concept for agricultural innovations in general.

## 2. Crop Breeding Innovations

Over the past decades, the genetic improvement of crops has contributed significantly to enhanced food security by increasing crop yield and resistance. Crop breeding innovation started with traditional breeding programs and, more recently, random mutagenesis using chemicals and irradiation, while the emergence of recombinant DNA technologies greatly expanded the potential for the genetic modification of crops. Genetic alteration of plants using recombinant DNA often relies on the use of *Agrobacterium*-mediated transformation, which results in the random insertion of T-DNA into the plant genome. The generation of a plant with a single copy of a transgene with a stable expression across environments requires extensive selection, screening, and evaluation over a number of generations. Modern genetic engineering approaches using these DNA technologies have been used since the 1990s to create a wide variety of improved crops. GM varieties of the large commodity crops, namely cotton, maize, soybean, and rapeseed, are currently the primary transgenic crops that are cultivated worldwide. The majority of these crops have been modified with herbicide and/or pest resistance traits, which only require the insertion of one or a few genes [12]. These traits can help to improve crop yield and lower costs by reducing the number of agrochemicals used on them.

In recent years, precise methods for gene editing have been developed, with CRISPR-Cas-based methods being the most prominent ones [13]. These methods allow for the specific and targeted engineering of DNA, and could decrease the time needed to create a genetically engineered plant with the “best” transgenic event. In addition, gene editing methods may be used to create small insertions, deletions, or substitutions that could theoretically also be the result of natural mutations or of random mutagenesis. More recent studies on the application of CRISPR-Cas methodology in crop plants show, however, that the approach may also lead to unintended off-target changes in the plant’s genetic make-up that are preventable through the design of the molecular tools and procedures applied [14,15].

Recent studies have demonstrated the specific modification of genes of interest in maize [14], rice [16], and soybean [17], among others. These studies show that CRISPR-Cas-based methods may be applied to achieve the effective expression of new desirable traits in a food crop through the modification of native genes or the introduction of new genes. Furthermore, Shi et al. (2017) succeeded in altering the expression of a native gene within GM maize variants by using CRISPR-Cas9 to insert a constitutive promoter in front of the coding sequence of the *ARGOS8* gene, which is associated with drought resilience, leading to increased yield under drought stress conditions [18].

Furthermore, advances in synthetic biology offer the possibility to use all available information, genetic or otherwise, to design and create biological factors, pathways, and networks for the improvement of crops [19,20,21,22].

## 3. Safe-by-Design in Agriculture

Safe-by-design principles are generally not yet implemented in the field of biological innovation, including crop breeding. There have, however, been developments in the field of nanomaterials. Kraegeloh et al. (2018) propose, based on different European projects, a comprehensive strategy for the use of the safe-by-design concept for the various fields of application of nanomaterials, ranging from electronics to drug delivery agents [23]. The authors also show a direct link between safe-by-design and RRI, with a focus on the following three aspects: functionality, safety, and communication. Four main dimensions are often described as the backbone of an RRI framework, namely:

1. Anticipation: considering the potential drawbacks and risks, but also the benefits of new technologies, thereby increasing resilience;

2. Inclusiveness: engagement of stakeholders, thereby including voices from society in research and innovation;

3. Reflexivity: reflecting on potential impacts on society and incorporating identified values into research and innovation; and

4. Responsiveness: the capacity to respond to societal needs and values, but also to new circumstances and surprises [24].

Through these dimensions, RRI takes into account the potential impact of the research and developmental processes and the related products on society at large, for instance, through early stakeholder involvement, as well as by promoting access to scientific results and aiming to create more value for users and society. Kraegeloh et al. (2018) further advocate the integration of these aspects for effective innovation on the basis of nanomaterials in a way that is safe for humans and the environment, and with better acceptance by society at large. For the latter, it is proposed to create a system of “trusted environments”, where innovators and regulators can have open communication lines on all of the aspects of innovation [23]. 

So far, for products of modern biotechnology, the regulatory assessment of the safety of GM foods and feeds for humans and animals is dealt with at a relatively late stage of product development, and is done on a non-standardized, case-by-case basis. Notably, various authorities have been proposed to consider the “familiarity” of a given modification if it has already been previously assessed in a number of instances. These safety aspects are part of the regulatory package provided to authorities when product developers request marketing approval for their product. This package currently comprises a comprehensive set of safety data, including details of the genetic modification; the changes that the modification has brought about at the molecular level, such as inserted DNA and newly expressed proteins; and the potential hazards linked with these changes. In more detail, the package usually contains a comprehensive analysis comparing the GM food/feed to a conventional non-GM counterpart with a history of safe use. This comparison commonly entails an extensive compositional analysis, covering nutrients, anti-nutrients, toxins, and other key components that are characteristic for the particular food or feed. Based on the differences identified, it is decided whether additional tests for, e.g., toxicity, allergenicity, or nutritional impact, are necessary in order to conclude on the safety of the product. The tests that have to be carried out straddle in-vitro, in-silico, and in-vivo testing, such as biochemical laboratory assays, bioinformatics-supported computer predictions, and animal trials, respectively. This is according to the comparative safety assessment approach, which has been internationally harmonized and became enshrined in the Food and Agriculture Organization (FAO) and World Health Organization’s (WHO) Joint Codex Alimentarius guidelines for the safety assessment of foods derived from microorganisms, plants, and animals using recombinant DNA techniques [25,26,27].

Owing to the current developments in this field, the time required for the research and development of gene-edited crops has decreased, and the technological thresholds have been lowered [28,29]. In order to be able to cope with the myriad of possible new or modified traits and number of applications, it is therefore necessary to “future-proof” the current approaches to maintain the safety of our food and feed supply, while avoiding the regulatory system getting overwhelmed. The safe-by-design approach appears fit for this purpose, as it facilitates the pre-market safety assessment of the final new crop plant products, by the continuous generation, collection, and evaluation of (safety) data throughout the research and development process. This will also allow for timely adjustments of the breeding program by considering the safety of the intermediate and final products. Besides the use of safe material, product, and production processes, the safe-by-design concept also comprises the safe use and end-of-life of products, hence covering the entire life cycle [30].

The safe-by-design concept may also prove valuable in the case of agricultural and related food innovations other than crop breeding, where it may likewise be applied. In the case of all agricultural innovations, the eventual goal is the same, to produce a safe end-product and to utilize available data in combination with adequate tools at each step of the research and development process, from the initial project idea through the design, research and development, and manufacturing, to the market release and possible post-market evaluations. Besides the safety of the target product for food and feed use, environmental safety aspects should also be included, for instance, the potential impacts on non-target organisms and gene flow to other species, which also extends to the management of waste products and potential by-products, or the transport of materials.

## 4. The Safe-by-Design Concept in Crop Breeding Innovation

**Figure 1:**
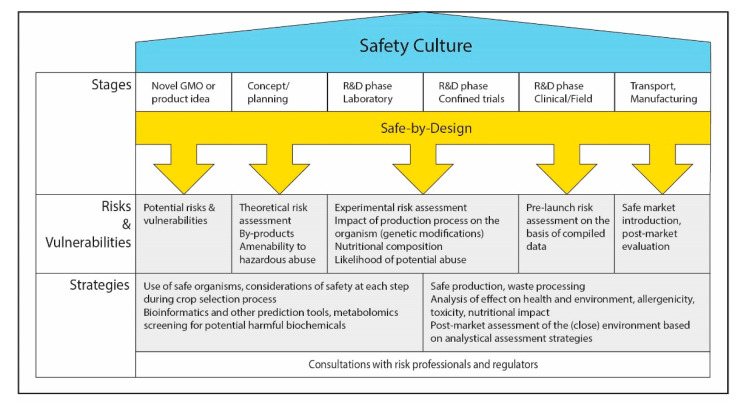
Safe-by-design concept in crop breeding: workflow from conceptualization of an idea until the release of the product on the market. Safe-by-design is an approach wherein safety evaluation is an inherent part of every stage of development, which may lead to the initiation of strategies to mitigate risks and vulnerabilities, and may ultimately contribute to the establishment of a safety culture.

For the development of novel agricultural products, we propose to apply the safe-by-design concept, as outlined in Figure 1, to ensure safety for the consumers and environment, by instilling awareness of safety aspects from the early design phases through to the final market release, such as the following:

1. Intended effects. Will the novel crop contain new or adjusted genetic elements? If so, what are the hazards involved in using this particular element?

2. Unintended effects. Hazards of (by-)products that may cause adverse effects, such as toxicogenic and allergenic compounds, produced by the new breed, or reduced nutritional characteristics. This may be caused by the genetic modification, or, more likely, by secondary trait effects.

In practice, this concept aims to reduce risks, where applicable, for instance through the utilization of safe organisms and well-characterized biological components in the creation of novel GM food and feed crops. Bioinformatics tools, as mentioned above, can be used to initially predict any unintended effects in the early design stages, and once a potential new crop has been generated, it may be screened, as part of the development, for harmful compounds through the use of, for instance, informative (metabol)omics screening methods. Most, if not all, of these aspects will already basically form part of current breeding programs, but when applying the safe-by-design concept, all choices made throughout the breeding programs, as well as all (agronomic and compositional) data obtained, will be assessed explicitly with relation to the safety of the product under development. The resulting crops will still require a safety assessment in each case, while the available knowledge on and familiarity with the host organism and introduced or altered components may facilitate the overall assessments and obviate the use of animal experimentation and other tests with the newly expressed products, amongst others.

From a broader perspective, safe-by-design would also serve as a key component of a positive organizational “safety and health culture”, being proactively promoted by various governments around the globe, and as such, a structural part of the educational programs of young scientists. Within such a culture, all parties within an organization are committed to safety based on shared perceptions and trust, with visible management actions toward that end, as well as proactive communication at all levels, transparency, employee participation, and controls and inspections [31]. As a case-in-point, “food safety culture” provides an interesting example from the related field of food production and processing. In the United Kingdom, for example, this concept has been adopted from the food industry’s Global Food Safety Initiative, and is incorporated into the British Retail Consortium’s Global Standard for Food Safety. This standard is followed worldwide by suppliers of food to the retail sector. Moreover, the national Food Standards Agency has incorporated elements into inspectors’ guidelines; while not accrediting the culture per se, various elements, such as management organization, strategies and long-term visions, awareness raising, compliance with standards, and investment in food safety, amongst others, are encouraged [32].

Besides the scientific safety aspects, as stated, this concept will fit in an RRI strategy, focusing on societal priorities and preferences by promoting stakeholder engagement [33]. A transparent concept, such as the proposed safe-by-design approach, including the safety aspect as a guiding principle from the onset of a research and development project, may benefit society and industry alike by resulting in safe products.

## 5. Consultations with Risk Assessors, Regulators, and the Public

Safe-by-design is a new way of including safety aspects throughout all stages of a research and developmental process. In this approach, the responsibility for the safety of the product to be marketed should rest primarily with the developer, who has the insight into its safety aspects. The actors involved in the development of innovative products should be endowed with the skills necessary to assess all of the available data at each stage in the development for possible consequences for the safety of intermediate and final products. At the same time, the developer should preferably be adequately supported by independent risk assessors throughout the entire procedure, although other stakeholders should also be involved when following an RRI strategy. Safety will thus become an inherent quality of a new product to be developed, instead of an aspect that receives attention only at the final stage of product development. In this way, the actors will have more insight into and will be continuously and proactively engaged with possible safety aspects of their innovative products, and can feed this information into a meaningful RRI route.

Public consultations, preferably in combination with stakeholder engagement events, are important in an RRI strategy that includes our proposed application of safe-by-design, as they promote transparency and may ultimately contribute to the adoption of a safety culture. Notably, Soeteman-Hernandez et al. (2019) also foresaw that a dialogue with stakeholders within a trusted environment would feed into the safe innovation approach, which combines safe-by-design elements with regulatory preparedness [6]. Examples of interactions between developers, regulators, and the public will be further analyzed by taking current consultation procedures of the United States, European Union, and Australia–New Zealand as examples.

Consultations with risk professionals are nowadays already in place. Such consultations may form part of a broader safe-by-design approach. In the USA, for instance, developers of novel plant varieties, including those developed using biotechnological techniques, have the opportunity to engage with the Food and Drug Administration (FDA), with the goal to ultimately bring a safe food product to the market [34]. The FDA encourages these voluntary informal consultations between developers and FDA risk assessors to obtain feedback early on during the developmental process regarding potential safety considerations, as well as information about the safety assessments. For transparency, concise reports of the completed consultations are made available to the public by the FDA in an online inventory. Similarly, in Australia and New Zealand, Food Standards Australia New Zealand (FSANZ) and the Office of the Gene Technology Regulator (OGTR) consult with the public regarding the intentional release of genetically modified organisms (GMOs), such as for the cultivation of novel GM crops [35,36]. The authorization process by the OGTR consists of, amongst others, consultations with relevant authorities such as FSANZ, governmental bodies, and expert committees. Following these consultations, a draft risk assessment and risk management plan is prepared and made available to the afore-mentioned groups and to the public, in order to provide feedback on the application.

In the European Union, new transparency rules will come into effect in March 2021, as per the recently adopted amendment to the General Food Law, through Regulation (EU) 2019/1381. These enable the consultation of the general public, which will have access to risk assessment studies submitted by applicants for the pre-market safety assessment, so that their completeness can be checked. In addition, applicants will have to register the studies they want to perform or commission for their application in advance, through a dedicated register. The European Commission will also have the possibility to organize fact-finding activities or, in exceptional cases, to commission research in order to verify risk assessment evidence. Vice versa, for those applications that will result in a scientific opinion, applicants will have the possibility to request advice from EFSA related to the notification. This EFSA advice is also to become visible for the general public. No EFSA advice is allowed to be given, though, on the design of specific dossier studies. Furthermore, the staff providing the advice must not be involved in preparatory scientific and technical work for a scientific opinion on the same product. More specifically, public consultations are also held on a regular basis, on the risk assessments completed by EFSA for the environmental release of GMOs (Directive 2001/18/EC) and GM foods and feeds (Regulation (EU) no. 1829/2003). During 30-day commenting periods, the public can submit comments through the European Commission’s website (https://ec.europa.eu/food/plant/gmo/public_consultations_en), and the collective comments received will be published on the website as well.

The above-mentioned public consultation approaches benefit transparency, but they all have in common that the consultations are held at the end of the development process, often after safety assessments have been performed by the authorities. From an RRI perspective, it would be preferred to involve relevant stakeholders at an earlier timepoint to allow for reflection and responsiveness to potential societal, ethical, and safety aspects.

## 6. Conclusions

The concept of safe-by-design has proven its value in manufacturing and other industries, but has not yet been applied in the agricultural sector for the development of novel food and feed products. Current advancements in agricultural innovation, however, do require new ways of thinking about adequate systems in order to guarantee the safety of our food supply and the environment in the long run. These innovations will primarily relate to the application of NBTs, including genome editing, and synthetic biology, which have the potential to play a significant role in future agricultural innovation.

The expected increase in the number of products with gene edited modifications that will move towards the world market may be difficult to cope with using the current procedures for regulatory approval, including a pre-market risk assessment and the related requirements for traceability. Given the concurrent safety concerns over new technological developments, it is recommended to consider a harmonized safe-by-design approach in modern agriculture that warrants the in-depth discussion of safety issues at each step of the development of a new agricultural product, from project idea through research and development, to the final product. Our description of the application of the safe-by-design concept in crop breeding could function as a basis for the development of a framework for crop breeding innovation. Such a safe-by-design framework should entail adequate interaction between product developers, risk professionals, and regulators, as well as the public at large through public engagement events and consultation, as part of a broader RRI approach. Taken together, this allows for a timely assessment of all insights and data generation throughout the research and development phase, thus offering additional assurance that the product being developed will ultimately pass the safety requirements for its market access in a way that will safeguard public health and create a win–win scenario for society and producers alike.

## Appendix A

**Table A1:** Examples of worldwide initiatives to ensure design through early measures during product or process development.

Terminology	Sector	Subject	Notes	Reference
System safety	General	This is an approach to eliminate hazards and reduce risks if such hazards cannot be eliminated System safety applies to a wide variety of items, such as equipment, systems, products, hardware, software, etc. throughout all stages of their lifecycle, starting from design and ultimately ending with disposal Roles and tasks are ascribed to stakeholders within the overall process, such as contractors, subcontractors, vendors, and suppliers. This procedure will also involve the establishment of product teams and working groups with various representatives reviewing hazard incident data, as well as agreeing on mitigation measures, amongst others	Hazards are initially identified and listed, the associated risks assessed, measures to mitigate these risks formulated, and risks reduced. Part of the System Safety approach is the preliminary hazard analysis (PHA), which is one of the various examples of hazard and risk assessment methods It will be assessed whether the hazard-associated risk is high, serious, medium, low or eliminated. Mitigation measures should aim at eliminating the hazard or, if this is not possible, to reduce risks to the lowest possible amount within the boundaries of financial, timing and performance constraints, for which a “hierarchy of controls” approach can be followed	[2,3,4]
Quality by Design	Pharmaceutical production	The targeted product’s risk attributes, including its critical ones, are to be defined already at an early stage of development by both developers and manufacturers A risk management strategy is to be elaborated on for product quality control throughout the life cycle Process performance criteria in the design of the production process itself are to be established, whilst the effectiveness of the control strategy needs to be verified continuously	“Quality by design” has become enshrined in internationally harmonised guidelines The “design space” has to be described, i.e., the variation that can be allowed in the process design, for example for adjustments during commercial manufacturing	[5]
Hygienic Engineering and Design	Food manufacture and processing	Design of food factories and manufacturing equipment with a focus on hygiene so that food safety and quality are not adversely affected	An extensive range of voluntary international guidelines has been published Facilities and equipment should be easy to clean so as to prevent food contamination, for example: ○ Contact surfaces: Avoid crevices and dead spaces where residues of food will stay behind, as a potential substrate for pathogenic microorganisms ○ Surfaces should not be made of toxic materials or that absorb substances that they are exposed to, as well as be resistant to breakage, leakage, or wear-off, so as to prevent the contamination of foods with chemicals and particles ○ Equipment should be impermeable to micro-organisms ○ Facilities should be designed to prevent the introgression of pests	[37]
Intrinsic safety	Electric engineering	In the design stage, care is taken to ensure that the wiring and other components of electric installations will not be able to initiate explosions, for example by sparks (arcs) or heat-producing elements strong enough to do so	Intrinsic safety has been incorporated into multiple standards of the IEC 60079 series of the International Electrotechnical Commission (IEC), as well as national electrotechnical regulations implementing these standards, such as the CENELEC standard and the ATEX Directive 94/9/EC in the EU. Besides the design stage, it also pays due consideration to, e.g., the installation, operation and maintenance of the equipment. Solely intrinsically safe items are allowed to be operated in the most hazardous areas	[38,39]
Intrinsic safety	Chemical processing	Identification of hazards through a “process hazard analysis” (such as “What if” or HAZOP), followed by the application of the “hierarchy of controls” Inherently safer approaches, including the following four items: Substitution: replace a hazardous chemical with a less dangerous material Minimisation: use and store smaller quantities of a hazardous chemical, and reduce the size of the equipment operating with these chemicals under risky conditions, such as high temperature or pressure Moderation: reduce risks by, for example, diluting, cooling, or differently processing hazardous chemicals Simplification: reduce unnecessary process complexity	The concept was initially articulated and promoted by the British chemical engineer T. Kletz in the late 1970s and became enshrined in, for example, guidance for chemical process safety issued by the American Institute of Chemical Engineers in the 1990s	[40,41]
Prevention through design	Occupational health	Inclusion of safety features into the design of items and workplaces that impact on workers in order to minimise hazards and risks, so as to prevent illness, injuries, accidents and fatalities, and to reduce risk to acceptable levels A holistic, systems approach is followed in addition to technical requirements, instilling a culture of safety and actively engaging various stakeholders in the process	In line with national and international standards, such as voluntary American National Standard ANSI/ASSP Z590.3, general safety management standard ANSI/ASSP Z10 for occupational safety and health, and ISO 45001 describing management systems’ structure for control and improvement of ergonomics Extending on successful examples from other sectors, such as nuclear energy, aviation, robotics, machinery, and semiconductor manufacture	[3,42,43]
Safe design	Construction	Safety arrangements for temporary and mobile construction site Safety and health are properly considered during preparation and execution of a construction project, and post-hoc use of the construct	Not only technical provisions but also the responsibility of staff and education of those involved Enshrined in regulations (*e.g.,* UK), governmental policies, and accreditation schemes (*e.g.,* Australia and USA)	[42,44,45]
Safe Design	Architecture	“Environmental crime reduction”, focusing on the criminological aspects of urban landscape architecture, building and interior design	Properties can be certified according to the a Canadian council’s SAFE Design Standard	[46]
Safer by design	Mining	Safety features of mobile equipment to be used in quarries, such as bulldozers, excavators, and dump trucks	Voluntary guidance for manufacturers and users	[47]
